# Deletion of AMPA receptor GluA1 subunit gene (*Gria1*) causes circadian rhythm disruption and aberrant responses to environmental cues

**DOI:** 10.1038/s41398-021-01690-3

**Published:** 2021-11-15

**Authors:** Gauri Ang, Laurence A. Brown, Shu K. E. Tam, Kay E. Davies, Russell G. Foster, Paul J. Harrison, Rolf Sprengel, Vladyslav V. Vyazovskiy, Peter L. Oliver, David M. Bannerman, Stuart N. Peirson

**Affiliations:** 1 Department of Physiology, Anatomy and Genetics, University of Oxford, Oxford, UK; 2 Department of Experimental Psychology, University of Oxford, Oxford, UK; 3 Sleep and Circadian Neuroscience Institute (SCNi), Nuffield Department of Clinical Neurosciences, University of Oxford, Oxford, UK; 4 IT Services, University of Oxford, Oxford, UK; 5 Department of Psychiatry, University of Oxford, Warneford Hospital, Oxford, UK; 6 Research Group of the Max Planck Institute for Medical Research at the Institute for Anatomy and Cell Biology, Heidelberg University, Heidelberg, Germany; 7 Mammalian Genetics Unit, MRC Harwell Institute, Harwell, UK

**Keywords:** Schizophrenia, Neuroscience

## Abstract

Dysfunction of the glutamate α-amino-3-hydroxy-5-methyl-4-isoxazolepropionic acid (AMPA) receptor GluA1 subunit and deficits in synaptic plasticity are implicated in schizophrenia and sleep and circadian rhythm disruption. To investigate the role of GluA1 in circadian and sleep behaviour, we used wheel-running, passive-infrared, and video-based home-cage activity monitoring to assess daily rest–activity profiles of GluA1-knockout mice (*Gria1*^−/−^). We showed that these mice displayed various circadian abnormalities, including misaligned, fragmented, and more variable rest–activity patterns. In addition, they showed heightened, but transient, behavioural arousal to light→dark and dark→light transitions, as well as attenuated nocturnal-light-induced activity suppression (negative masking). In the hypothalamic suprachiasmatic nuclei (SCN), nocturnal-light-induced cFos signals (a molecular marker of neuronal activity in the preceding ~1–2 h) were attenuated, indicating reduced light sensitivity in the SCN. However, there was no change in the neuroanatomical distribution of expression levels of two neuropeptides―vasoactive intestinal peptide (VIP) and arginine vasopressin (AVP)―differentially expressed in the core (ventromedial) vs. shell (dorsolateral) SCN subregions and both are known to be important for neuronal synchronisation within the SCN and circadian rhythmicity. In the motor cortex (area M1/M2), there was increased inter-individual variability in cFos levels during the evening period, mirroring the increased inter-individual variability in locomotor activity under nocturnal light. Finally, in the spontaneous odour recognition task GluA1 knockouts’ short-term memory was impaired due to enhanced attention to the recently encountered familiar odour. These abnormalities due to altered AMPA-receptor-mediated signalling resemble and may contribute to sleep and circadian rhythm disruption and attentional deficits in different modalities in schizophrenia.

## Introduction

Sleep and circadian rhythm disruption (SCRD) is reported frequently in neuropsychiatric diseases and is observed in up to 80% of schizophrenia patients [1–3]. A clearer picture of the range of sleep disturbances in schizophrenia is now emerging, including sleep fragmentation, and reductions in total sleep time and rapid eye movement sleep latency. Unstable and fragmented rest–activity rhythms are also reported, indicating there is also a strong circadian component to daily behaviour that can be disrupted in this disorder. While it is accepted that drug treatments are likely to influence these specific factors, it is becoming increasingly apparent that the comorbidity of SCRD and schizophrenia may be due to the disruption of common anatomical morphology or signalling mechanisms in the brain [4, 5].

Of the neurotransmitter systems implicated in neuropsychiatric diseases, the evidence for an aetiological role for glutamate dysfunction in schizophrenia is well-supported both at pathological and genetic levels [6–8]. From the latest genome-wide association studies of schizophrenia, glutamate receptor loci have been consistently identified as being associated with the disorder; for example, a particular locus within 500 kb of the *Gria1* gene (hg19 position, chr5:151941104–152797656) is highly genome-wide significant (*p* = 1.055 × 10^−10^) [9]. *Gria1* encodes the GluA1 (also known as GluR-A or GluR1) subunit of the α-amino-3-hydroxy-5-methyl-4-isoxazolepropionic acid (AMPA) subtype of glutamate receptors. GluA1-containing AMPA receptors are important for synaptic plasticity; for example, the GluA1 subunit mediates rapid AMPA receptor trafficking after long-term potentiation induction [10]. Moreover, levels of GluA1 and other glutamatergic transcripts are reduced in post-mortem brain tissues of schizophrenia patients [11–13].

The cognitive and sensorimotor gating abilities of GluA1 knockout mice have been explored in multiple studies [14–24]. They exhibit short-term attentional deficits, impaired pre-pulse inhibition, and novelty-induced hyperlocomotion―all behavioural abnormalities considered relevant to the positive symptoms of schizophrenia [25–28]. At the physiological level, these cognitive and learning deficits are correlated with reduced hippocampal synaptic transmission [29–31] and impaired synaptic plasticity in the hippocampal synapses [14, 30–32]. Without GluA1, mice show unregulated attentional processing of the environment, as indicated by persistently heightened levels of θ-band (6–12 Hz) power in the dorsal hippocampus and prefrontal cortex [33]. The GluA1-knockout mouse therefore provides a valuable model for studying glutamatergic dysfunction and deficits in synaptic plasticity implicated in neuropsychiatric diseases.

GluA1 is also implicated in the homeostatic regulation of sleep and wakefulness, although its precise role remains to be determined. For example, GluA1-containing AMPA receptor levels in the postsynaptic density of the neocortex and hippocampus of rats are elevated after an extended period of spontaneous wakefulness and decrease after a period of consolidated sleep [34, 35]. In addition, GluA1 levels in the cortex and hippocampus increase after sleep deprivation, suggesting a functional link between synaptic GluA1 levels and the re-setting of synaptic weights due to sleep pressure [34]. In addition, our recent study has reported a marked reduction in sleep electroencephalogram (EEG) spindle activity in GluA1 knockout mice [36]―another characteristic feature of schizophrenia [2].

Glutamatergic signalling is also crucial for light perception and regulation of circadian rhythms. Glutamate is the primary excitatory neurotransmitter of the retinohypothalamic tract, by which light signals from the retina are conveyed to the master circadian clock in the hypothalamic suprachiasmatic nuclei (SCN) [37–39]. Whilst *N*-methyl-d-asparate (NMDA) receptors play a key role in these processes [40, 41], non-NMDA AMPA receptors also contribute to photic and circadian responses [42–44]. *Gria1* mRNA is expressed in bipolar, amacrine, and retinal ganglion cells (RGCs) in the human retina [45], and GluA1 proteins are localised to the inner plexiform layer and amacrine and ganglion cell bodies of the rat retina [46]. GluA1 proteins and mRNA are expressed in the dorsal (shell) and ventral (core) subregions of the mouse SCN [42, 43] and are also widely distributed in the hamster SCN [47]. Crucially, glutamatergic neurotransmission also plays a role in SCN efferent and afferent pathways [48], and AMPA receptors mediate interhemispheric SCN neuronal synchronisation [44].

As circadian disturbances have been well characterised in schizophrenia, studying the consequences of disrupted AMPA -receptor-mediated glutamatergic signalling may provide new insights into the mechanistic basis of SCRD in neuropsychiatric diseases [4, 49]. Accordingly, we used running wheels, video tracking, and passive-infrared sensor (PIR) recording to examine circadian and sleep behaviour in GluA1-knockout mice. In our recent study, we have validated that ≥40 s of PIR inactivity is correlated with EEG-defined sleep, with Pearson’s *r* ≥ 0.95 [50]. Thus, PIR recording allows high-throughput screening of circadian rhythms and sleep patterns in a non-invasive manner, avoiding unnecessary surgery-related confounding effects (e.g., local neuroinflammatory responses caused by EEG electrode implantation [51]). In addition, we examined SCN and motor cortical area M1/M2 cFos signals in response to nocturnal light [52], as well as the expression of two main neuropeptides in the SCN, namely vasoactive intestinal peptide (VIP) and arginine vasopressin (AVP). SCN cells expressing these neuropeptides receive synaptic inputs from melanopsin-expressing photosensitive RGCs (pRGCs) and can be directly excited by light; [53] both neuropeptides are known to be important for neuronal synchronisation within the SCN and circadian rhythmicity [54, 55]. Finally, we assessed GluA1 knockouts’ short-term memory performance using the spontaneous recognition memory task [56], which is known to be sensitive to SCRD [57–59] and GluA1 deficiency [17, 22]. Instead of using the standard object and visuospatial tasks, here we used the odour task [60] to see if GluA1 knockouts would show aberrant responses in a task where stimulus discrimination did not rely on retinal input. We conducted odour recognition testing at midday and midnight, to see if GluA1 deficiency would lead to a phase change in the optimal time of performance or impair performance equally in both light and dark phases.

## Methods

### Animals

A total of 19 male GluA1 knockouts (*Gria1*^−/−^) and 19 male wild-type littermates on a C57BL/6J background were used in behavioural experiments (cohort 1–3); ages of these mice are summarised in Table 1. Female mice were not used, as oestrous cycles affect activity rhythms [61] and sleep propensity [62]. A separate cohort of six GluA1 knockouts and 6 wild-type littermates was used for immunohistochemistry (cohort 4); ages of cohort 4 were within the age range of cohorts 1–3. Genetic construction, breeding, and genotyping of GluA1 knockouts (B6N.129-*Gria1*^*tm1Rsp*^/J; Mouse Genome Informatics ID: MGI:2178057) was as previously described [14]. The genotype of each animal was known to experimenters before the start of each experiment. Experiments were carried out in accordance with the United Kingdom Animal [Scientific Procedures] Act 1986, with procedures reviewed by the clinical medicine animal welfare and ethical review body (AWERB) and conducted under project licences PPL 30/3353, PPL 30/3371, and PPL 30/2812. Mice were provided with food and water ad libitum and maintained under 12-h light/12-h dark cycles (12:12 LD; 200-lux cool-white LED light, measured at the cage floor). They were singly housed in light-tight chambers (six cages per chamber). To minimise the contribution of any extraneous environmental factors to behavioural rhythms, wild-types and GluA1-knockout animals were alternated and evenly spaced within each light-tight chamber.

**Table 1:** Cohorts of mice used for circadian screening.

Cohort	*n* (WT/KO)	Start age (days)	End age (days)	Wheels	*Tau* in DD/LL wheels	Video-tracking	PIR	Bouts	IS/IV	*Tau* in DD/LL PIR
1	7/7	118–183	177–242	✓	✓					
2	7/5	57– 121	101–165			✓	✓	✓	✓	
3	5^a^/7	101–168	224–291			✓	✓	✓	✓	✓
Totals	19/19			7/7	7/7	12/12	11/12	11/12	11/12	4/7

^a^One wild-type mouse was found dead before the completion of circadian screening.

### Circadian screening

We used three different ways to assess home cage activity, including wheel-running, video tracking, and PIR recording. Table 1 summarises the number of GluA1 knockouts and wild-types in each of the three cohorts.

#### Cohort 1 (running wheels)

Cages equipped with running wheels (Colborn Instruments) were used for circadian screening of cohort 1. Mice were entrained for 21 days under 12:12 LD, with Zeitgeber time (ZT) 0 starting at 7 am. This was followed by a jet-lag protocol of 6-h phase advance of light onset, with 14 days under the new 12:12 LD schedule (ZT0 starting at 1 am). After 2 weeks of re-entrainment, a 1-h nocturnal light pulse was given at ZT16. The extent of nocturnal-light-induced phase shift (∆*φ*) was examined by comparing activity onsets under 6 days of LD before the light pulse vs. activity onsets under 6 days of constant dark (DD) after the light pulse (the Aschoff type II protocol). In addition, to assess endogenous free-running activity rhythms mice were placed under constant dark (DD) and constant light (LL) for at least 7 days. The number of wheel rotations was recorded continuously using ClockLab (Actimetrics).

#### Cohorts 2 and 3 (no running wheel)

Circadian and immobility-determined sleep data were obtained as previously described [50, 63–65]. Video tracking data were obtained from cohorts 2 and 3. Three independent 24-h video recordings were taken 7 days apart during 21 days under 12:12 LD. These video files were replayed and automated tracking of locomotor activity was conducted in ANY–maze (version 4.5; Stoelting) [63]. Locomotor activity data were also obtained from cohorts 2 and 3 using PIRs (Panasonic, AMN32111 NaPiOn) [50]. Activation of the PIR was detected every 100 ms (% time active reported at 10-s intervals). For both video tracking and PIR activity data, immobility-defined sleep was defined as ≥40 s of inactivity [50, 63]. To examine negative masking and sleep induction in response to nocturnal light, 1-h light pulses were presented from ZT14 to ZT15; light pulses were presented on three different nights, separated by a week of baseline 12:12 LD recording.

### Activity/sleep bouts and measures of circadian rhythm stability

A bout of activity was defined as any one or more consecutive 10-s bins in which the mouse was active. The intensity of activity in the bout is the percentage of the entire bout that the sensor is active. For example, six consecutive bins with activity would give a bout length of one min and a 50% intensity would mean the sensor was active for 30 s of that 1 min. If the sensor detected no movement for four consecutive bins, a bout of sleep was recorded (starting with the fourth 10-s bin), as ≥40 s of immobility has been shown to correlate highly with EEG-defined sleep in mice [50, 63]. The stability of circadian rhythms was assessed using established measures of intraday variability (IV) and interday stability (IS) [66]. Bouts of activity and sleep, as well as measures of stability in the actigraphy, were calculated using the same seven days of PIR recordings that were used to construct the 24-h pattern of activity. Circadian characteristics of daily activity, including period length and oscillatory power of ~24-h rhythms, were quantified by generating *χ*^2^ periodograms using ActogramJ (version 0.9) [67].

### Nocturnal light pulses and lighting transitions at dusk and dawn

One-hour light pulses were presented from ZT14 to ZT15; light, in order to examine sleep induction by nocturnal light. Light pulses were presented on three different nights, separated by a week of baseline 12:12 LD recording in the interim. For each mouse, daily profiles for activity and immobility sleep over three light pulses were generated for ZT13–16 in one-min bins, as were the baseline profiles for ZT13 – 16 from the seven days prior to the first nocturnal light pulse. Average activity and sleep in one-min bins were similarly generated for ZT23 to ZT1 (dark → light transition) and ZT11 to ZT13 (light → dark transition) from the seven days prior to the first nocturnal light pulse.

### Immunohistochemical staining for cFos, VIP, and AVP

Half of wild-types and GluA1-knockout mice did not receive any light pulse prior to perfusion; the remaining animals were perfused ~90 min after the onset of nocturnal light (starting from ZT14). Mice from both dark and light-pulse conditions were injected intraperitoneally with sodium pentobarbitone at ~ZT15.5 (±30 min). Immediately after the loss of the pedal reflex, they were perfused transcardially with phosphate-buffered saline (PBS) and subsequently with 4% paraformaldehyde (PFA). Mouse heads were fixed in 4% PFA overnight, and brains were extracted and kept in PFA the next day. On the third day, brains were rinsed with PBS and transferred into 30% sucrose PBS for cryopreservation. Three days later, brains were sectioned coronally at 50 μm on a freezing microtome (Leica). Free-floating coronal sections were stored in PBS with 0.1% sodium azide. Coronal sections containing the optic chiasm and SCN (5–7 sections per brain) from all four conditions were processed on the same days to minimise variability in immunostaining quality. Sections were washed in PBS and subsequently blocked with normal donkey serum (Jackson ImmunoResearch, 017-000-121-JIR) for 90 min. They were then incubated with guinea pig anti-cFos (Synaptic Systems, 226 005; dilution 1:2000), rabbit anti-VIP (Invitrogen, PA5-78224; dilution 1:500), and mouse anti-AVP (Santa Cruz Biotechnology, sc-390723; dilution 1:1000) at room temperature on the first day and at 4 °C on the second and third days. After 72 h of primary-antibody incubation, sections were washed in PBS and incubated with Alexa Fluor 488 donkey anti-guinea pig, Cy3 donkey anti-rabbit, and Alexa Fluor 647 donkey anti-mouse secondary antibodies (Jackson ImmunoResearch, 706-545-148-JIR, 711-165-152-JIR, and 715-605-151-JIR, respectively; dilution 1:500 for all three secondary antibodies). After 4 h of secondary-antibody incubation, sections were washed in PBS and mounted on glass slides and cover slipped with Vectashield (Vector Laboratories, H-1000). Z-stack images of the SCN and motor cortex (area M1/M2) were acquired under a ×20 objective lens using the Olympus Fluoview FV1000 confocal microscope; identical settings were used for scanning samples from all four conditions. Immunofluorescence signals were quantified in Fiji ImageJ [68].

### Short-term odour recognition memory performance

Mice from cohort 3 received two spontaneous odour recognition memory trials at ZT6 (±30 min) and two trials at ZT18 (±30 min) inside a 20 cm × 20 cm × 20 cm open-top white acrylic arena. A camera was positioned ~40 cm above the centre of the arena. The arena and camera were housed inside a light-tight wooden chamber equipped with cool-white LEDs, providing a light level of ~100 lux at both midday and midnight. Each odour recognition trial consisted of a ≤5-min sample phase, a three-min delay interval, and a one-min test phase. Only 1 trial was given every 12 h. Eight essential oils were selected as odour stimuli; these included: lemon, vanilla, and peppermint extracts (Dr. Oetker); orange, chocolate, and rose extracts (Nielsen-Massey); and banana and jasmine essence (Double Seahorse). A different pair of odour stimuli was used as familiar and novel odours on each trial. Prior to the sample phase, 1 mL of each odour stimulus was delivered with a syringe into an amber glass vial (base diameter 1.5 cm × height 2.2 cm); there were multiple replicates of the glass vial, and different replicates were used in sample and test phases for each mouse. During the sample phase of each trial, the mouse freely explored two glass vials containing the sample odour cue. The sample phase was ended after the mouse spent 40 s in odour exploration or after 5 min elapsed (whichever came first). Three minutes later, in the test phase the mouse freely explored a glass vial containing the sample odour cue and a glass vial containing an entirely novel odour cue for 1 min. Automated tracking of the mouse’s exploration was conducted in ANY-maze (version 4.5; Stoelting). The floor of the arena (20 cm × 20 cm) and two circular notional zones (diameter 5 cm) centred at the location of the glass vials were defined in ANY-maze. The position of the mouse’s snout was tracked and recorded every 0.1 s. The total amount of time spent inside each of the two notional zones was determined. Odour recognition memory performance was calculated as the percentage of time spent with the novel odour cue; a score of 50% indicates no discrimination between novel and familiar odour cues.

### Data analyses

Statistical analyses were conducted in GraphPad Prism (version 7, GraphPad Software) and SPSS version 22 (IBM). Parametric analyses including independent sample *t*-tests and split-plot analyses of variance (ANOVAs) were conducted to examine genotype effects. In split-plot ANOVAs, Time of Day (ZT0–23) was entered as the within-subjects factor and Genotype as the between-subjects factor; Greenhouse–Geisser corrections were applied to the degrees of freedom in cases where the assumption of sphericity was violated. Means ± standard errors of the mean were reported in the Results section and plotted in figures; asterisks in figures represent significant differences: ****p ≤ 0.0001; ***p ≤ 0.001; **p ≤ 0.01; *p < 0.05.

## Results

### Rest–activity rhythms under 12:12 LD

**Fig. 1:**
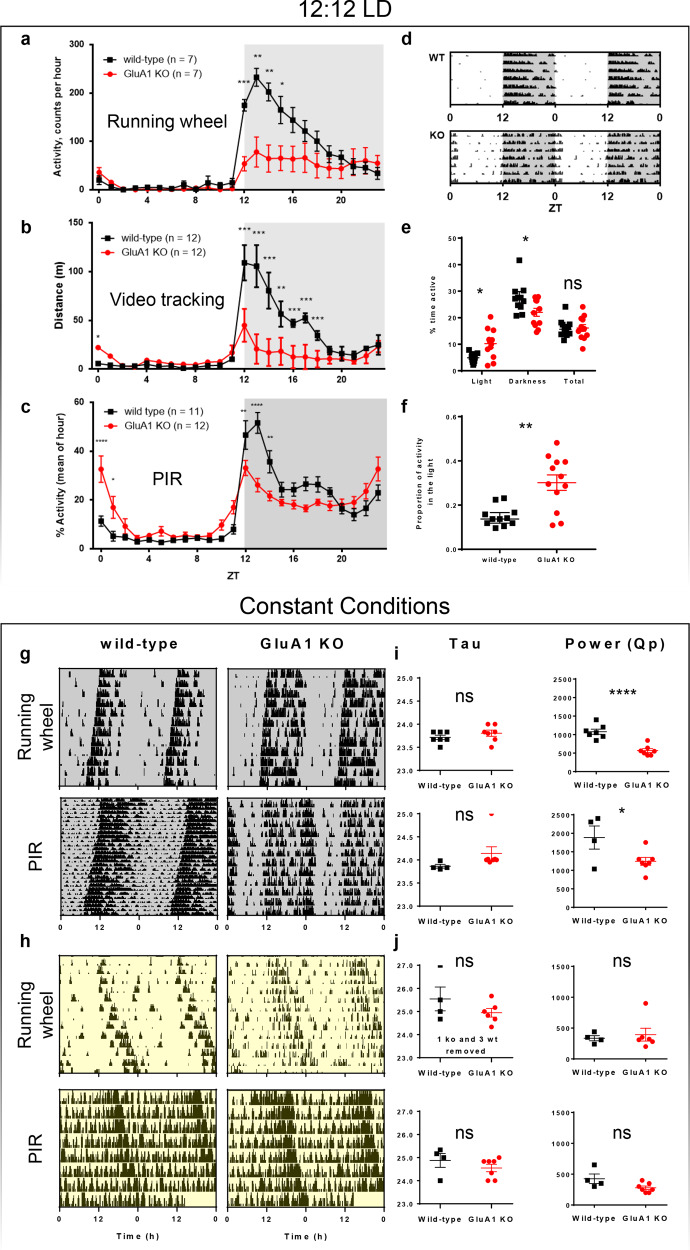
Rest–activity rhythms under 12:12 LD and constant conditions. **a**–**f** Actigraphy data from GluA1-knockout mice and wild-type littermates under 12:12 LD. **a** 24-h distribution of activity over 12 days of 12:12 LD, as defined by revolutions of a running wheel. Similar distributions of activity over 24 h, as recorded using other methods of assessing activity: **b** distance moved in video recordings and **c** total activity assessed by PIR sensors. **d** Example double-plotted actograms show seven days of wheel-running activity from a representative wild-type mouse and a representative GluA1-knockout mouse, showing that the GluA1-knockout mouse’s activity offset was extended into the light phase. **e** Overall daily PIR activity and average % activity in light and dark phases. **f** Light-phase PIR activity expressed as a proportion of total PIR activity. Values in **e** and **f** are from seven days of PIR recording shown in **c**. **g**–**j** Circadian rhythm parameters under constant conditions. **g**, **h** Examples actograms collected using running wheels and PIR under constant darkness (DD) and constant light (LL, 200 lux), respectively. **i**, **j** Circadian period (*tau*; in hours) of the predominant periodicity and size of this peak derived from *χ*^2^ periodograms (power, Qp) under DD and LL, respectively. DD (running wheels): *n* = 7 wild-types, *n* = 7 GluA1 KO, 14 days. DD(PIR): *n* = 4 wild-types, *n* = 7 GluA1 KO, 30 days. LL (running wheels): *n* = 7 wild-types, *n* = 7 GluA1 KO, 14 days, three wild-types and 1 GluA1 KO lacking significant rhythmicity. LL(PIR): *n* = 4 wild-types, *n* = 7 GluA1 KO, 10 days. ****p ≤ 0.0001; ***p ≤ 0.001; **p ≤ 0.01; *p < 0.05.

To assess daily activity and circadian entrainment, GluA1 knockout mice and littermate controls were housed under 12:12 LD. Our previous work has shown that wheel-running activity ― a complex behaviour modulated by motor and motivational processes ― may not always reflect actual daily locomotor activity [65]. As such, we used three different ways to assess home cage activity, including wheel-running, video tracking, and PIR recording. Data from all three methods showed that GluA1 knockouts displayed consistent changes in daily activity (Fig. 1a–c).

Analyses of wheel-running data (Fig. 1a) showed that the overall wheel-running activity level was slightly lower in the GluA1 knockout mice [main effect of Genotype: *F* (1, 11) = 4.43, *p* = 0.059]. In addition, there was a significant interaction between Genotype and Time of Day [*F* (23, 253) = 7.53, *p* < 0.001], primarily because GluA1 knockouts were less active in the dark phase (Fig. 1a and Table 2). Post hoc testing (Bonferroni corrected) showed that wild-types ran more on the wheel than GluA1 knockouts at night from ZT12 to ZT16 (*p*s < 0.024). Despite having reduced wheel-running activity, GluA1 knockouts exhibited a daily active period (*alpha*) of wheel-running activity ~3 h longer than that of wild-type mice (Table 2).

**Table 2:** Additional circadian parameters of GluA1 knockouts assessed under running wheels.

12:12 LD	WT	GluA1 KO	*p* value
Total daily (24-h) activity [*Wr*]	14998 ± 1661	7706 ± 2863	0.059
Dark phase (12-h) activity [*Wr*]	14858 ± 1632	7623 ± 2830	0.058
Light phase (12-h) activity [*Wr*]	140 ± 35	83 ± 35	0.277
Daily active period, *alpha* [h]	9.66 ± 0.62	12.79 ± 0.52	0.002**
**DD**	**WT**	**GluA1 KO**	***p*** **value**
Total daily (24-h) daily activity [*Wr*]	14915 ± 2155	5294 ± 1759	0.005**
Daily active period, *alpha* [h]	10.49 ± 0.38	13.51 ± 0.59	0.001**
**LL**	**WT**	**GluA1 KO**	***p*** **value**
Total daily (24-h) daily activity [*Wr*]	6549 ± 1528	2727 ± 1032	0.06

Descriptive statistics (means ± standard errors of the mean) for selected circadian parameters. Units of measurement are indicated in square brackets.

*Wr* number of wheel rotations.

**significant genotype differences from independent sample *t*-tests (*ps* < 0.01).

Analyses of video-tracking data (Fig. 1b) and PIR data (Fig. 1c) also revealed significant interaction effects between Genotype and Time of Day on locomotor activity [*video tracking*: *F* (23, 460) = 19.60, *p* < 0.001; *PIR*: *F* (23, 483) = 43.07, *p* < 0.0001]. Consistent with the wheel-running data, GluA1 knockouts were less active at night (Bonferroni-corrected post hoc testing: *video tracking* from ZT12 to ZT19 all *p*s < 0.05; *PIR* from ZT12 to ZT15 all *p*s < 0.005). However, in contrast to the wheel-running data, both video-tracking and PIR analyses indicated that GluA1 knockouts were more active at the start of the day (Bonferroni-corrected post hoc testing: *video tracking* from ZT0 to ZT1 *p* = 0.021; *PIR* from ZT0 to 2; both *p*s < 0.05). In addition, similar to the lengthened active period of wheel running, the daily active period of PIR activity was ~4 h longer in GluA1 knockouts (*alpha* = 12.00 ± 0.58 h vs. 16.04 ± 0.38 h; *p* < 0.0005), extending several hours into the next day. Thus, there was a misalignment of daily activity with respect to the light/dark cycle in GluA1 knockouts (see also the delayed wheel-running activity offset in Fig. 1d).

As PIR sensors are the most sensitive to circadian abnormalities, especially the increase in activity at the start of the light phase (Fig. 1a–c), further analyses were conducted specifically on PIR data. The average intensity of PIR activity in GluA1 knockouts was higher in the light phase, but lower during the dark phase, resulting in no significant difference between genotypes when total activity was considered (Fig. 1e). The proportion of light-phase activity has been widely used as a marker of circadian disruption, and this was found to be significantly increased in GluA1 knockouts (Fig. 1f). These data are consistent with GluA1 knockouts’ altered sleep EEG patterns in our previous study, which found increased wakefulness EEG in the light phase [36].

GluA1 knockouts’ extended active period but reduced total wheel-running activity may seem paradoxical, but it could be related to changes in overall circadian parameters, for example reduced robustness of the ~24-h rhythm; it could also be the result of altered microstructure of activity and rest [69], such as changes in activity/sleep bout length due to activity/sleep fragmentation. These possibilities were thoroughly investigated below.

### Rest–activity rhythms under constant conditions

To assess the endogenous circadian clock of GluA1 knockouts, wheel running and PIR data were studied under constant dark (DD) and constant light (LL) conditions. Example actograms in Fig. 1g, h show expected period shortening and lengthening of activity rhythms under DD and LL, respectively; this is reflected in the average free-running period length (*tau*), which is ≤24 h under DD and >24 h under LL (Fig. 1i, j).

Under DD, no significant difference in *tau* of GluA1 knockouts was observed compared to wild-types with running wheels [*t* (12) = 1.01, *p* = 0.292]; similarly, no difference in *tau* was observed under PIR sensors [*t* (6.929) = 1.862, *p* = 0.105; Fig. 1i]. Both methods of assessment showed significantly attenuated rhythm robustness in GluA1 knockouts, as defined by the size of the peak harmonic (Qp) in the *χ*^2^ periodogram [*running wheel*: *t* (12) = 5.881, *p* < 0.0001; *PIR*: *t* (9) = 2.396, *p* = 0.0402; Fig. 1i]. Under DD, GluA1 knockouts also exhibited an active period of wheel-running activity ~3 h longer than that of wild-type mice, despite the fact that GluA1 knockouts’ daily wheel rotations were less than half of wild-types’ daily wheel rotations (Table 2).

Although some animals lacked rhythmicity under constant light (LL), the value of *tau* of the remaining mice could be calculated, and this was not significantly different between genotypes [*running wheel*: *t* (8) = 1.273, *p* = 0.239; *PIR*: *t* (9) = 1.063, *p* = 0.315; Fig. 1j]. In addition, there was no significant difference in the size of Qp in the *χ*^2^ periodogram under LL [*running wheel*: *t* (8) = 0.437, *p* = 0.674; *PIR*: *t* (9) = 2.145, *p* = 0.061; Fig. 1j]. Daily wheel rotations under LL were not significantly different between genotypes (Table 2).

With no significant difference in free-running periods, but reduced *χ*^2^ periodogram power under DD, these data suggest that circadian rhythms are generally preserved in GluA1 knockout mice, but they are less robust than wild-type animals.

### Intraday variability and interday stability of PIR activity rhythms

**Fig. 2:**
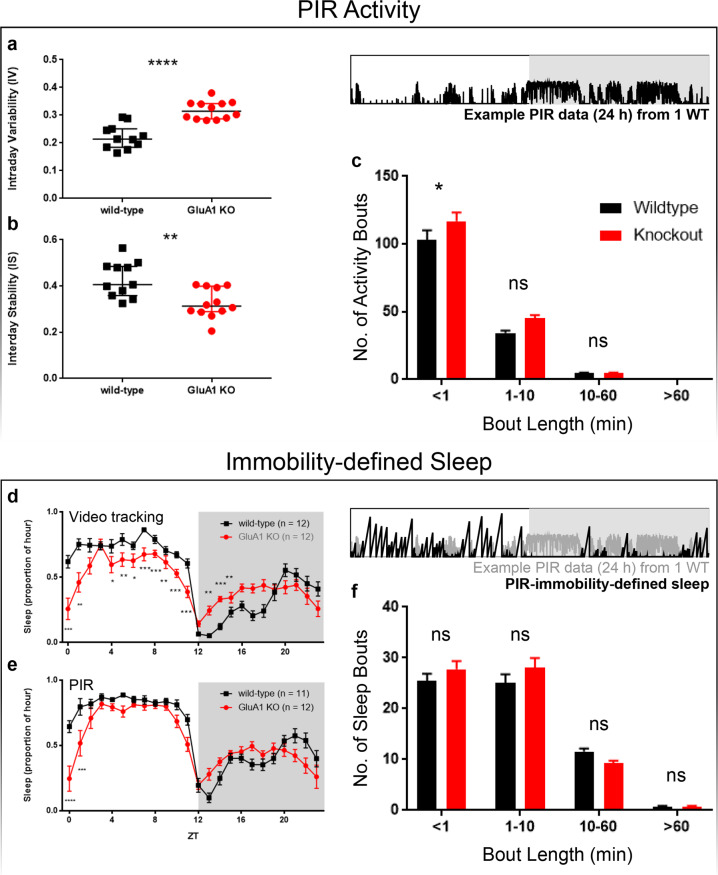
PIR activity bouts and immobility-defined sleep. **a**–**c** PIR activity. An example PIR recording (24 h) from one animal is shown in the black trace. **a** Interday stability (IS) of PIR activity, from seven days of 12:12 LD activity data. **b** Intraday variability (IV) of PIR activity, from the same seven days of 12:12 LD activity data. **c** Numbers of short (<1 min) and long PIR activity bouts (≥1 min) observed in wild-type (*n* = 11) and GluA1 KO (*n* = 12) mice from PIR data over seven days of 12:12 LD. GluA1 knockouts showed more short activity bouts (<1 min) than wild-type mice. **d**–**f** Behavioural sleep. An example PIR recording (24 h) is shown in the grey trace and PIR-immobility-defined sleep is shown in the black trace. **d** Behavioural sleep extracted from *video-tracking* data: 24-h distribution of average hourly proportion of sleep over three non-sequential days of 12:12 LD, as defined by ≥40 s of immobility under video tracking. **e** Behavioural sleep extracted from *PIR* data: 24-h distribution of average hourly proportion of sleep over 7 sequential days of 12:12 LD, as defined by ≥40 s of PIR inactivity. **f** Numbers of short (<1 min) and long immobility-defined sleep bouts (≥1 min) observed in wild-type (*n* = 11) and GluA1 KO (*n* = 12) mice from PIR data over seven days of 12:12 LD. ****p ≤ 0.0001; ***p ≤ 0.001; **p ≤ 0.01; *p < 0.05.

Increased variability and fragmentation of the 24-h activity profile have been well-documented in patients with schizophrenia;[70, 71] therefore, further analyses of PIR activity and sleep were carried out, using the seven days of 12:12 LD PIR data for detailed measures of stability and bout analysis. Static measures of stability with actigraphy have focused on either the number of transitions from activity to rest (intraday variability, IV), or the consistency of a pattern of activity and rest from one day to the next (interday stability, IS). GluA1-knockout mice were less stable in terms of both measures. These data demonstrated a significant increase in IV, main effect of genotype *F* (1, 19) = 30.57, *p* < 0.0001, as well as a decrease in IS, main effect of genotype *F* (1, 19) = 10.52, *p* = 0.0043, in GluA1 knockouts (Fig. 2a, b, respectively).

#### Activity bouts

Differences in the distribution of PIR activity bouts were also observed between GluA1 knockouts and wild-types. Mutants displayed short (<1 min) bouts of activity more frequently than wild-types; however, there was no difference in the frequency of longer (≥1 min) bouts of activity (Fig. 2c).

### Immobility-defined sleep

Sleep under 12:12 LD was assessed from three days of video tracking (Fig. 2d) as well as seven days of PIR recording (Fig. 2e) using validated behavioural parameters [50, 63]. For each 1-s bin of the video-tracking data and each 10-s bin of the PIR data, the mouse’s behavioural state was assigned as either awake (0) or asleep (1), where “1” was defined as sustained immobility for at least 40 s; if this was not the case, a value of “0” was assigned. Sleep proportion (Fig. 2d, e) was then determined as the number of bins assigned with a value of “1” divided by the total number of 10-s bins in 1 h (i.e. 360 bins). Sleep bouts of different lengths (Fig. 2f) were tallied up: <1 min (<6 bins), 1–10 min (6–60 bins), 10–60 min (60–360 bins), and >60 min (>360 bins). Our previous studies have validated that in mice ≥40 s of behavioural immobility is highly correlated with EEG-defined sleep, with Pearson’s *r* ≥ 0.95 [50, 63].

GluA1 knockouts spent less total time asleep than wild-types when assessed by video-tracking [main effect of Genotype *F* (1, 20) = 5.46, *p* = 0.030]. Importantly, there was a significant interaction between Genotype and Time of Day [*F* (23, 460) = 7.85, *p* < 0.001], due to the fact that GluA1 knockouts slept less during the light phase but more at night from ZT13 to ZT15 (Bonferroni-corrected post hoc testing all *p*s < 0.05; Fig. 2d). These sleep data from video tracking seemed to be broadly consistent with immobility-defined sleep obtained from PIR data, with GluA1 knockouts sleeping less during the light phase but more at the beginning of the night (Fig. 2e); however, the size of the genotype effect was smaller under PIR recording. Thus, sleep timing in GluA1 knockouts is distributed differently from wild-type mice, with less sleep at the start of the day and more sleep at night. These results are partly consistent with GluA1 knockouts’ altered sleep EEG patterns in our previous study, which reported delayed sleep onset and fewer NREM and REM sleep episodes in the light phase [36].

#### Sleep bouts

As more fragmented activity bouts were observed in GluA1 knockouts, the distribution of immobility-defined sleep bouts was also examined. Interestingly, despite the increased number of short activity bouts in GluA1 knockouts (Fig. 2c), frequencies of short (<1 min) and long sleep bouts (≥1 min) were indistinguishable between genotypes (Fig. 2f).

### Phase advance of wheel-running activity under jet lag and phase delay induced by nocturnal light

During the assessment of wheel-running activity, an abrupt 6-h phase advance of 12:12 LD was applied. We used wheel-running activity onset as the phase marker to assess re-entrainment under jet lag. Mean ± standard errors of the mean phases of the onset of wheel-running activity (relative to ZT12 under the new LD) across the seven days following the abrupt 6-h phase advance were: 17.10 ± 0.55 h, 16.43 ± 0.53 h, 15.31 ± 0.51 h, 14.34 ± 0.94 h, 13.09 ± 1.28 h, 12.64 ± 0.98 h, and 12.10 ± 1.03 h in wild-types; and 16.61 ± 0.58 h, 15.97 ± 0.65 h, 15.42 ± 0.68 h, 14.55 ± 0.58 h, 13.09 ± 0.93 h, 12.18 ± 0.57 h, and 11.38 ± 0.74 h in GluA1 knockouts. These were statistically indistinguishable between genotypes [main effect of Genotype *F* (1, 12) = 2.33, *p* = 0.153; Genotype × Day interaction *F* (15, 180) = 0.81, *p* = 0.669]. For each mouse the rate of re-entrainment under jet lag was estimated from the slope (*m*) of the least-squares linear regression line, activity onset = *m* × days + constant. Mean rates of re-entrainment were −0.89 ± 0.058 h day^−1^ in wild-types and −0.91 ± 0.031 h day^−1^ in GluA1 knockouts, indicating that both genotypes advanced their wheel-running rhythms ~1 h a day; these were not different from each other (*p* = 0.670). Under the Aschoff type II nocturnal light protocol, the onset of wheel-running activity was delayed in both genotypes: ∆*φ* = +0.38 ± 0.34 h in wild-types and +0.33 ± 0.33 h in GluA1 knockouts; these were not different from each other (*p* = 0.932). Thus, circadian photoentrainment of wheel-running activity was unaffected in GluA1 knockouts.

### Changes in locomotor activity and sleep in response to nocturnal light

**Fig. 3:**
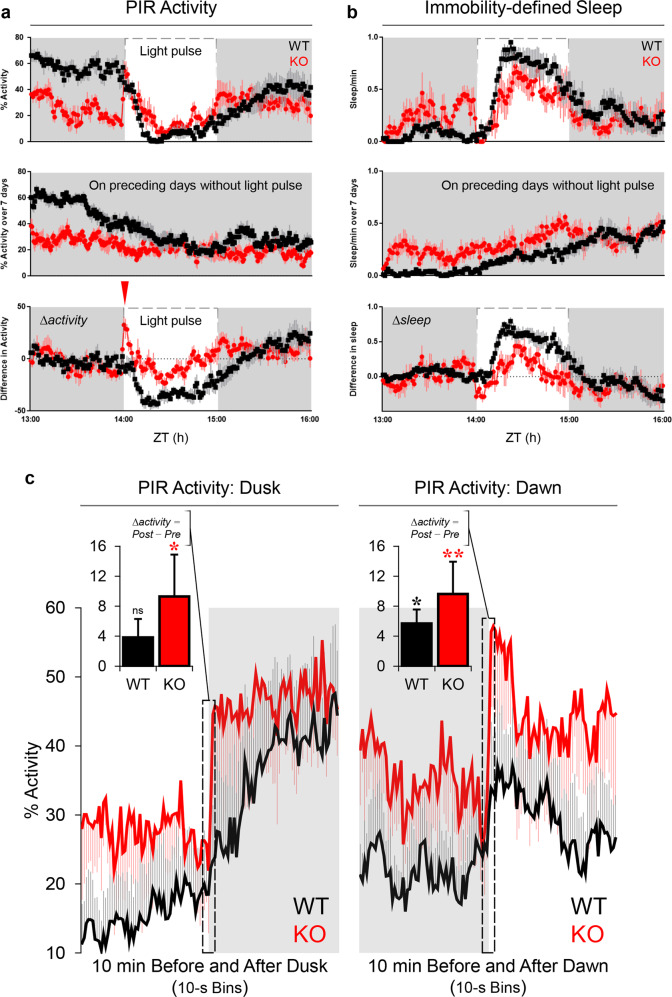
Behavioural responses to nocturnal light pulses and to dusk and dawn transitions. **a** PIR activity (averaged across three replicates, as 1-min bins) in response to a 1-h light pulse, which is indicated by the dashed line. Overall activity (top) compared to average activity at the same time of day (ZT13–16) on the seven days prior to the 1st light pulse (middle), to give the change in activity (∆activity) in response to the light pulse (bottom). The red inverted triangle indicates the sharp but transient spike of PIR activity immediately following the onset of the light pulse in GluA1 knockouts. **b** Similar examination of changes in the proportion of time spent asleep, where sleep was defined as ≥40 s of PIR inactivity. Light-induced sleep was attenuated in GluA1 knockouts (main effect of Genotype on ∆sleep from ZT14–15, *p* < 0.05; bottom). **c** PIR activity changes in response to dusk (i.e. the transition from light to dark; left) and dawn (i.e. the transition from dark to light; right). Data were pooled across 8 days of 12:12 LD. Changes in locomotor activity at dusk and dawn were exaggerated in GluA1-knockout animals (two min before vs. two min after dusk: Genotype × Time Bins interaction *p* = 0.004; main effect of Genotype *p* = 0.343; two min before vs. two min after dawn: Genotype × Time Bins interaction *p* < 0.001; main effect of Genotype *p* = 0.267). **c**, *insets* In wild-type animals (black) only small and gradual changes in locomotor activity were observed at dusk and dawn (10 s before vs. 10 s after dusk: mean ∆activity = +3.84%, *p* = 0.169; 10 s before vs. 10 s after dawn: mean ∆activity = +5.70%, *p* = 0.023; black asterisk). By contrast, sharper and exaggerated responses to dusk and dawn were found in GluA1-knockout animals (red; 10 s before vs. 10 s after dusk: mean ∆activity = +9.30%, *p* = 0.02; red asterisk; 10 s before vs. 10 s after dawn: mean ∆activity = +9.63%, *p* = 0.008; double red asterisks). A Genotype × Phase (Dusk and Dawn) ANOVA showed a main effect of Genotype on ∆activity scores (*p* = 0.037), confirming the increased sensitivity to light/dark transitions in GluA1-knockout animals.

To examine negative masking and sleep induction in response to nocturnal light, mice received a 1-h light pulse from ZT14 to ZT15―a time when mice﻿ show a high level of activity. In wild-type mice, the nocturnal light pulse supressed PIR activity (negative masking; Fig. 3a, top) and induced sleep (Fig. 3b, top). After adjustment for baseline activity (Fig. 3b, middle), light-induced sleep was still evident (Fig. 3b, bottom), consistent with our previous study [72]. By contrast, GluA1 knockouts responded differently to nocturnal light; this may have been partly due to genotype differences in baseline activity (Fig. 3a, middle). More specifically, light onset at ZT14 caused a sharp but transient spike of PIR activity in GluA1 knockouts (Fig. 3a, bottom). As locomotor activity can be viewed as a behavioural readout of arousal [73–76], our results suggest that GluA1 knockouts showed an exaggerated level of behavioural arousal in response to light onset, attenuating sleep induction/maintenance during the 1-h nocturnal light (main effect of Genotype on ∆sleep at ZT14–15, *p* < 0.05; Fig. 3b, bottom).

In agreement with the PIR data, video tracking data showed that, in wild-type mice, the 1-h nocturnal light suppressed 91.55% (±2.26%) of total activity relative to the previous night and induced 39.46 min (±2.49 min) of sleep; but in GluA1-knockout animals nocturnal light caused 65.71% (±5.87%) of activity suppression relative to the previous night [wild-types vs. GluA1 knockouts: *F* (1, 22) = 16.853, *p* < 0.001] and induced 25.44 min (±3.77 min) of sleep [wild-types vs. GluA1 knockouts: *F* (1, 22) = 9.626, *p* = 0.005]. In addition to attenuated negative masking and light-induced sleep, the GluA1-knockout group showed higher variability in locomotor activity under nocturnal light: standard errors of mean activity suppression by light = 2.93% in wild-types vs. 21.05% in GluA1 knockouts (Levene’s test for equality of variances *F* = 19.869, *p* < 0.001; Supplementary Fig. S1).

### PIR activity changes at dusk and dawn

To further explore this transient response to changes in lighting, we analysed PIR activity shortly before vs. after dusk and dawn transitions under 12:12 LD (Fig. 3c). Interestingly, changes in locomotor activity at dusk and dawn were exaggerated in GluA1-knockout animals [two min before vs. two min after dusk: Genotype × Time Bins interaction *F* (23, 230) = 2.038, *p* = 0.004; main effect of Genotype *F* (1, 10) = 0.989, *p* = 0.343; two min before vs. two min after dawn: Genotype × Time Bins interaction *F* (23, 230) = 2.677, *p* < 0.001; main effect of Genotype *F* (1, 10) = 1.384, *p* = 0.267]. More specifically, in wild-type animals only small and gradual changes in locomotor activity were observed at dusk and dawn (10 s before vs. 10 s after dusk: mean ∆activity = +3.84%, *p* = 0.169; 10 s before vs. 10 s after dawn: mean ∆activity = +5.70%, *p* = 0.023). By contrast, sharper and exaggerated responses to dusk and dawn were found in GluA1-knockout animals (10 s before vs. 10 s after dusk: mean ∆activity = +9.30%, *p* = 0.02; 10 s before vs. 10 s after dawn: mean ∆activity = +9.63%, *p* = 0.008; Fig. 3c, *insets*). Increased sensitivity to light/dark transitions is further confirmed by a Genotype × Phase (Dusk and Dawn) ANOVA conducted on ∆activity scores, which showed a main effect of Genotype [*F* (1, 10) = 5.817, *p* = 0.037]; the mean ∆activity in the 10 s after light/dark transitions was nearly doubled in knockouts (wild-types vs. GluA1 knockouts = +4.77 ± 1.34% vs. +9.46 ± 1.33%). Thus, GluA1-knockout mice exhibited increased sensitivity to lighting transitions.

### Functional readout of SCN activity and its chemical neuroanatomy

**Fig. 4:**
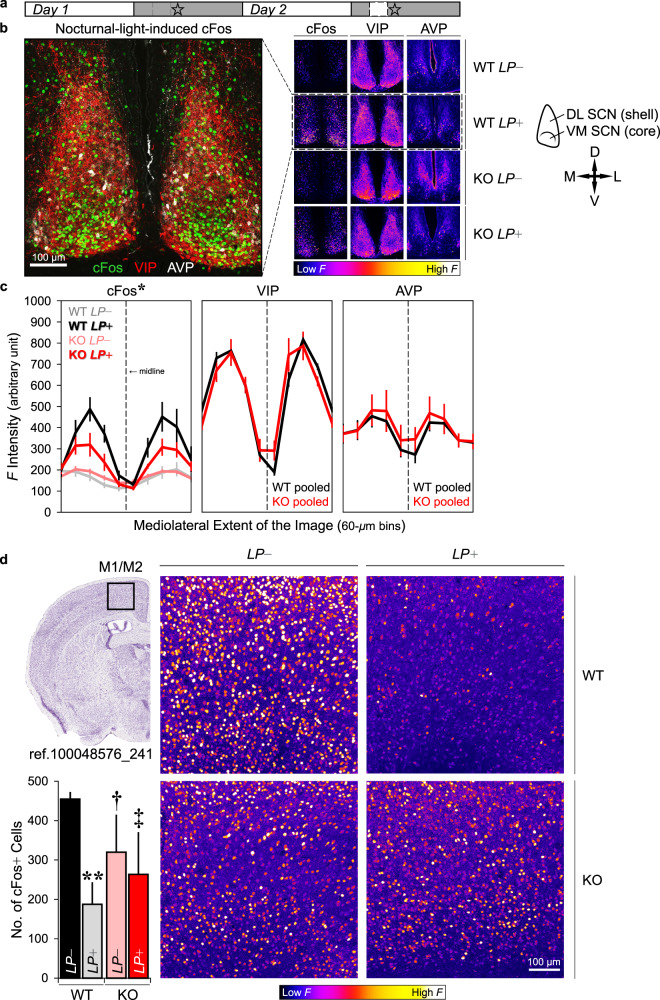
Immunofluorescence cFos, VIP, and AVP signals. **a** A schematic depicting the timeline of the experiment: For half of the animals (*n* = 3 wild-type and three GluA1 KO mice), no light pulse was given prior to perfusion and they were perfused at ~ZT15.5 (day 1, *LP*−); the remaining animals perfused on day 2 (*n* = 3 wild-type and three GluA1 KO mice) received a nocturnal light pulse (*LP*+) which started at ZT14. Star symbols indicate the Zeitgeber time of perfusion, which was ~90 min after the onset of the nocturnal light (~ZT15.5). Thus, the ZTs at which perfusion was performed were matched between *LP*− and *LP*+ conditions. **b** Representative images of immunofluorescence cFos, VIP, and AVP staining in the SCN (scale bar = 100 μm). In the middle multi-panels, brighter colours indicate higher immunofluorescence (*F*) intensity, whereas darker colours indicate lower *F* intensity. A schematic delineating the core (ventromedial) vs. shell (dorsolateral) subregions of the SCN is shown on the right (D dorsal, V ventral, M medial, and L lateral). **c** Mean *F* intensities (±standard errors of the mean) from all three channels across the entire mediolateral extent of the SCN. Nocturnal-light-induced cFos signals were attenuated in GluA1 knockouts (*two-way ANOVA for *LP*+ conditions, Genotype × Mediolateral Extent interaction *p* = 0.019). VIP and AVP data were pooled across *LP*− and + *LP* (*n* = 6 per genotype). The vertical dashed lines represent the location of the midline, and the 60-μm bins adjacent to the dashed lines (i.e. the middle two bins with the lowest *F* intensities) are the location of the 3^rd^ ventricle. **d** Example images of immunofluorescence cFos staining in the motor cortex, area M1/M2 (scale bar = 100 μm). The image of the Nissl-stained coronal section on the left was retrieved from the Allen Mouse Brain Atlas, plate reference number 100048576_241 [125]. The number of cFos+ cells in M1/M2 of wild-type animals, but not GluA1 knockouts, was reduced in response to nocturnal light (**p < 0.01). Variability in cFos+ cell count in KO *LP*− and KO *LP*+ conditions (pink and red bars) was higher than that in the WT *LP*− condition (black bar), as indicated by Levene’s tests for equality of variances (†*p* < 0.05; ‡0.05 < *p* < 0.06).

GluA1-knockout animals' circadian abnormalities and changes in behavioural responses to light could be the result of altered light input to the SCN or compromised SCN function (or both), which could be indicated by changes in: (1) immediate-early gene cFos, a molecular marker of recent neuronal activity; [52] or (2) neuropeptides that are known to be important for neuronal synchronisation within the SCN and circadian rhythmicity [54, 55], such as VIP and AVP that are differentially expressed in the core (ventromedial) vs. shell (dorsolateral) subregions of the SCN [53]. To assess functional changes, in a separate cohort of wild-type and GluA1-knockout mice we quantified immunofluorescence cFos signals in the SCN and motor area M1/M2, ~90 min following the onset of nocturnal light (started at ZT14); cFos expression in response to nocturnal light in the *LP*+ group was compared with the expression level at the same ZT (~ZT15.5) without any light pulse in the *LP*− group (Fig. 4a). In addition, to examine if there were any chemical neuroanatomical changes in the SCN, we quantified immunofluorescence VIP and AVP signals from the same brain sections.

Representative images of immunofluorescence cFos, VIP, and AVP staining in the SCN are shown in Fig. 4b, and immunofluorescence intensities from all three channels across the entire mediolateral extent of the SCN are summarised in Fig. 4c. In the absence of light (*LP*−), cFos signals were relatively low at night and there was no difference between genotypes [Genotype × Mediolateral Extent interaction *F* (9, 36) = 1.555, *p* = 0.166; Fig. 4c, left panel]. Nocturnal light (*LP*+) activated SCN cells in both wild-type and GluA1-knockout mice, as indicated by the main effect of Light Pulse [*F* (1, 8) = 17.720, *p* = 0.003]. Crucially, nocturnal-light-induced cFos signals were attenuated in the SCN of GluA1-knockout mice [Genotype × Light Pulse × Mediolateral Extent interaction *F* (9, 72) = 3.111, *p* = 0.003; *LP*+ groups: Genotype × Mediolateral Extent interaction *F* (9, 36) = 2.629, *p* = 0.019; Fig. 4c].

As no genotype difference was found in *LP*− groups, *LP*+ groups’ cFos intensity values were then divided by *LP*− group averages in the corresponding mediolateral bins, to take into account the baseline cFos intensity in the dark (ratio > 1 indicates *LP*+ > *LP*−; ratio ≈ 1 indicates *LP*+ ≈ *LP*−). The mean normalised cFos intensity was higher in the wild-type *LP*+ group than in the GluA1-knockout *LP*+ group [*F* (1, 4) = 9.512, *p* = 0.037; in the two 60-μm bins adjacent to the 3^rd^ ventricle]. Furthermore, one sample *t*-tests showed that the mean normalised intensity in the wild-type *LP*+ group, 2.535 ± 0.304, was significantly different from the value of 1 [*t*(2) = 5.046, *p* = 0.037 (2-tailed)], indicating a ~2-fold increase in SCN cFos expression. By contrast, the mean normalised intensity in the GluA1-knockout *LP*+ group, 1.491 ± 0.148, was not different from 1 [*t*(2) = 3.309, *p* = 0.080 (2-tailed)], confirming that cFos induction was attenuated in GluA1-knockout mice.

Neuroanatomical distribution of neuropeptides VIP and AVP in the SCN of both wild-type and GluA1-knockout mice were consistent with previously published results [53]. VIP staining was observed across the entire dorsolateral extent of the SCN. Noticeably, there was a narrow band of VIP+ cell bodies along the ventromedial border of the core subregion (Fig. 4b) near the optic chiasm; the rest of the SCN (shell) contained primarily VIP+ fibres but very few VIP+ cell bodies. By contrast, AVP+ cell bodies were found predominantly in the shell subregion of the SCN (Fig. 4b). VIP and AVP signals were statistically indistinguishable between genotypes [*VIP*: main effect of Genotype *F* (1, 10) = 0.010, *p* = 0.924; Genotype × Mediolateral Extent interaction Greenhouse‒Geisser *F* (2, 23) = 1.092, *p* = 0.361; *AVP*: main effect of Genotype *F* (1, 10) = 0.197, *p* = 0.667; Genotype × Mediolateral Extent interaction Greenhouse‒Geisser *F* (2, 27) = 0.710, *p* = 0.544; Fig. 4c, middle and right panels].

### Motor cortex, area M1/M2

Example images of immunofluorescence cFos staining in motor area M1/M2 are shown in Fig. 4d. Area M1/M2 of wild-type mice, but not of GluA1 knockouts, was strongly activated in the dark (*LP*−) but was relatively silent under *LP*+ [*WT*: *t*(4) = 4.733, *p* = 0.009; *KO*: *t*(4) = 0.398, *p* = 0.711; Fig. 4d]―corresponding to the strong negative masking response in wild-type mice and attenuated negative masking in GluA1-knockout mice (Supplementary Fig. S1). The lack of a difference between *LP*− and *LP*+ in GluA1 knockouts could be attributed to increased variability in cFos levels in the dark (WT *LP*− vs. KO *LP*−, Levene’s test for equality of variances *F* = 9.928, *p* = 0.034) and in response to nocturnal light (WT *LP*− vs. KO *LP*+, Levene’s test for equality of variances F = 7.209, p = 0.055). The increased inter-individual variability in motor cortical cFos levels during the evening period mirrored the increased inter-individual variability in locomotor activity under nocturnal light (Supplementary Fig. S1).

### Short-term odour memory process

In our previous studies [17, 22], we reported that GluA1-knockout animals showed impaired short-term visuospatial and object recognition memory performance; however, in these studies behavioural testing was conducted in the light phase only. Given that: (*1*) short-term recognition memory performance is known to exhibit day/night differences; [77] and (*2*) GluA1 knockouts’ rest–activity rhythms are misaligned with respect to the light/dark cycle (Figs. 1, 2), the optimal time for short-term memory processing could be shifted in these animals. To assess this possibility, each mouse received two spontaneous recognition memory trials at midday (ZT6) and two trials at midnight (ZT18). This allows us to see if GluA1 deficiency would lead to a phase change in the optimal time of performance or impair performance equally in both light and dark phases. In addition, we used the odour task [60], instead of the standard object and visuospatial tasks [17, 22], to see if GluA1 knockouts would show aberrant responses in a task where stimulus discrimination did not rely on retinal input.

**Fig. 5:**
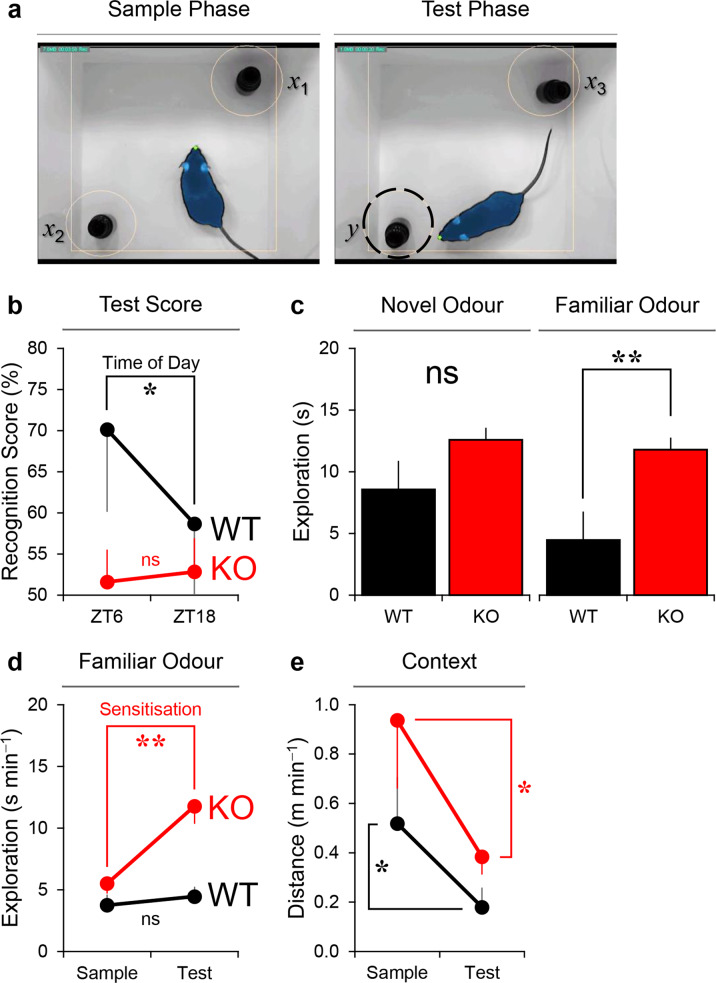
Impaired short-term odour memory performance in GluA1-knockout mice. **a** Screenshots from ANY–maze tracking. *x*_1_, *x*_2_, and *x*_3_ denote the three identical replicates of the glass vial containing the sample (familiar) odour, and *y* denotes the glass vial containing the novel odour at test. The shape of the mouse body as detected by ANY–maze is shaded in blue and the tip of its nose is marked by the green dot. **b** In wild-type mice (*n* = 5), odour recognition memory performance, expressed as [*y* ÷ (*x* + *y*)] × 100%, was better at ZT6 than at ZT18 (* one-way ANOVA, main effect of Time of Day *p* = 0.015). Test performance in wild-type animals was above chance level at ZT6 [one sample *t*-test comparing mean vs. 50%, *p* = 0.043 (1-tailed)] but not at ZT18 (*p* = 0.221). By contrast, in GluA1 knockouts (*n* = 7) recognition memory performance was at ~50% at both times of day (one-way ANOVA, main effect of Time of Day *p* = 0.866; one sample *t*-tests both *p*s > 0.25), indicating that GluA1-knockout mice failed to differentiate between novel and familiar odour cues in both light and dark phases. **c** The actual amount of time spent exploring the novel odour cue (in seconds) was not significantly different between groups (one-way ANOVA, main effect of Genotype *p* = 0.074, left panel). However, GluA1 knockouts showed increased exploration of the familiar odour cue at test (** one-way ANOVA, main effect of Genotype *p* = 0.002, right panel). **d** In wild-type animals, the rate of exploration in seconds min^−1^ towards the familiar odour remained unchanged from sample to test phases; whereas GluA1 knockouts showed an increased rate of exploration (Genotype × Sample/Test Phase interaction *p* = 0.001; ** simple effect of Sample/Test Phase in KO *p* = 0.001), indicative of sensitisation to the familiar odour over time. **e** In both genotypes, distance travelled in metres min^−1^ decreased from sample to test phases (* two-way ANOVA main effect of Sample/Test Phase *p* = 0.025; main effect of Genotype *p* = 0.149); this could be the result of motor fatigue rather than habituation to the background context [74].

During the sample phase of each trial, the mouse freely explored two glass vials containing the same odour cue (Fig. 5a). The sample phase was ended either after the mouse spent 40 s in odour exploration or after 5 min elapsed (whichever came first). Although there was some indication that GluA1 knockouts were more active in terms of odour exploration (expressed in seconds min^−1^) and distance travelled (expressed in metres min^−1^) during the sample phase―similar to that reported in our previous study [22]―these effects did not reach significance in the current experiment: odour exploration in wild-types vs. GluA1 knockouts = 3.75 ± 0.72 s min^−1^ vs. 5.51 ± 0.77 s min^−1^ [*F* (1, 10) = 2.555, *p* = 0.141]; distance travelled in wild-types vs. GluA1 knockouts = 0.52 ± 0.17 m min^−1^ vs. 0.94 ± 0.27 m min^−1^ [*F* (1, 10) = 1.380, *p* = 0.267].

In the test phase, the mouse freely explored a glass vial containing sample (familiar) odour cue and a glass vial containing an entirely novel odour cue; the test phase lasted for 1 min as in a previous study [60]. Odour recognition memory performance was calculated as the percentage of time spent with the novel odour cue at test. In wild-type mice, memory performance was better at ZT6 than at ZT18 [70 ± 9 % vs. 59 ± 10 % of time spent with novel odours at test; main effect of Time of Day *F* (1, 4) = 9.969, *p* = 0.034; Fig. 5b], in agreement with our previously published findings [77]. Test performance in wild-type mice was above chance level at ZT6 [one sample *t*-test (1-tailed), *p* = 0.043] but not at ZT18 (*p* = 0.221). By contrast, GluA1 knockouts’ recognition memory performance was at ~50% at both times of day [52 ± 4 % vs. 53 ± 4 % of time spent with novel odours at ZT6 and ZT18, respectively; main effect of Time of Day *F* (1, 6) = 2.218, *p* = 0.187; one sample *t*-tests (1-tailed) both *p*s > 0.25], indicating that GluA1 knockouts failed to differentiate between familiar and novel odour stimuli. Crucially, impaired memory performance was a result of GluA1 knockouts’ elevated exploration of the familiar odour cue in the test phase [Fig. 5c: main effect of Genotype *F* (1, 10) = 17.944, *p* = 0.002; Fig. 5d: Genotype × Sample/Test Phase interaction *F* (1, 10) = 20.223, *p* = 0.001; simple effect of Sample/Test Phase in GluA1 knockouts *p* = 0.001; simple effect of Sample/Test Phase in wild-types *p* = 0.179]. Aberrant salience of the familiar cue was negatively related to discrimination performance in GluA1 knockouts: the correlation between familiar odour exploration and recognition scores while controlling for novel odour exploration was −0.895 (*p* = 0.016). Distance travelled (m min^−1^) decreased from sample to test phases in both genotypes (Fig. 5e); this could simply be an indication of motor fatigue rather than (stimulus-specific) habituation to the background context [74].

## Discussion

The AMPA receptor GluA1 subunit has been studied extensively in many aspects of neurobiology and behaviour, and has been implicated in schizophrenia [25–28]. GluA1 knockouts have been consistently documented to be hyperactive during short-term exposure to a novel environment [16, 18, 20, 21]. However, these mice have been reported to show normal overall locomotion in home cage conditions, either by pressure-pad measurement in the dark phase [20] or over 24 h using a photobeam system [21]. In contrast to these previous studies, we observed reduced locomotor activity and increased immobility-defined sleep at night, but increased activity and reduced sleep at the start of the day. This is likely to be the result of higher sensitivity of video tracking and PIR systems used in the current study. Notably, GluA1 knockouts’ daily activity patterns in the current study are broadly consistent with their altered sleep EEG patterns in our previous study, which reported increased wakefulness EEG, delayed sleep onset, as well as fewer NREM and REM sleep episodes in the light phase [36].

### Circadian disturbance in comparison to patients and another mouse model

Circadian disruption can be defined in different ways, including misalignment between the internal biological clock and external environment, as well as desynchrony between molecular clocks in different tissues and cells throughout the body [78–80]. Such disturbances do not typically result in changes in circadian period. Instead, the robustness of the ~24-h rhythm is typically reduced, becoming increasingly fragmented and less stable. Circadian disturbance can be quantified using different metrics, such as increased light-phase activity, reduced periodogram power, increased intraday variability and reduced interday stability, as well as an increase in the number of activity bouts [79]. GluA1-deficient mice showed consistent changes in all of these metrics, indicating reduced robustness of their circadian rhythms and potentially mirroring the sleep and circadian disruption reported in schizophrenic patients [70, 71]. Some similarities in sleep and circadian abnormalities between patients and GluA1-deficient mice are summarised in Table 3. Circadian disturbances could be the result of altered AMPA-receptor-mediated signalling at multiple levels along the retina→SCN pathway (see Potential mechanisms below).

**Table 3:** Circadian and behavioural abnormalities in schizophrenia and GluA1 knockouts.

Schizophrenia patients	GluA1-deficient mice
1. Rest–activity rhythms misaligned with LD (*W*)	1. Rest–activity rhythms misaligned with LD (Fig. 1)^a^
2. Increased arousal episodes during bed time (*T*)	2. Increased activity during the resting phase (Fig. 1)^a^
3. Circadian period ≠24 h (*W*)	3. Reduced power of ~24-h rhythms (Fig. 1)
4. Increased day-to-day variability in sleep and melatonin onset, midpoint, and offset (*T*,*W*)	4. Increased day-to-day variability in rest–activity rhythms (Fig. 2)
5. Fragmented sleep–wake patterns (*T*,*W*)	5. Fragmented activity bouts (Fig. 2)
6. Deficits in sensorimotor gating (*B*)	6. Aberrant salience of recently encountered familiar﻿ environmental cues (Figs. 3,5)

*T* Tandon et al. [70], *W* Wulff et al. [71], *B* Braff and Geyer [93].

^a^Results 1 and 2 mirror GluA1 knockouts’ altered sleep EEG patterns in our previous study, which found increased wakefulness EEG, delayed sleep onset, as well as fewer NREM and REM sleep episodes in the light phase [36].

Previously, we reported compromised SCN function in another mouse model, *blind-drunk* (*Bdr*) mice with a mutation in the synaptosomal-associated protein 25, which leads to impaired synaptic vesicle exocytosis and schizophrenia-related phenotypes [81, 82]. In fact, the circadian phenotype of GluA1-knockout mice is in many ways comparable to that observed in the *Bdr* mutant mouse; this includes: (1) misaligned rest–activity rhythms to the external light environment; (2) increased light-phase activity; (3) reduced *χ*^2^ periodogram power; and (4) increased intraday variability and reduced interday stability; [82] GluA1 knockouts also showed increased variability in cFos levels in the motor cortex during the evening period, which could be related to increased PIR-activity variability. In our previous study we suggested that the circadian phenotype of *Bdr* mice was related to the phase advance of SCN AVP rhythm, disrupting synaptic communication between SCN neurons and altering SCN efferent signals to downstream regions [82]. This may not be the case for GluA1-knockout mice, because unlike *Bdr* mice, there was no change in SCN AVP expression in GluA1 knockouts during the evening period. However, a full time-course investigation across the diurnal cycle is required in future studies to confirm if the phasing of AVP expression is altered in GluA1 knockouts.

### Potential mechanisms

Fragmented and less robust rhythms in GluA1-knockout mice could be due to perturbed circadian rhythm generation in the central clock in the SCN. In addition, attenuated negative masking and SCN cFos induction could reflect reduced sensitivity to light at the level of the SCN. Whilst additional data are required to disentangle the relative contribution of GluA1-mediated signalling at the level of the retina vs. SCN, it is likely that changes can occur at multiple levels along the retina→SCN pathway in GluA1-knockout mice. For example: (1) Altered responses to light could occur at the level of the retina, as GluA1 is expressed in bipolar cells, amacrine cells, and RGCs in the retina [45, 46] and signalling from bipolar cells onto RGCs involves AMPA receptors [83]. (2) Changes could occur at the pRGC→SCN synapse. In the mouse SCN GluA1 proteins and mRNA are differentially expressed across the core and shell subregions, with higher levels of expression in the shell subregion [42, 43]. As AVP-expressing cells in the shell subregion receive synaptic inputs from pRGCs and can be directly excited by light [53], GluA1 deficiency at the pRGC→SCN AVP+ cell synapse may have affected responses to light. (3) As interhemispheric communication between the left and right SCN can be blocked by AMPA receptor antagonists [44], GluA1 deficiency may have affected signalling between SCN cells. (4) AMPA-receptor-mediated signalling between the SCN and its postsynaptic targets may also be affected, such as the organum vasculosum lamina terminalis (OVLT) [84] and hypothalamic paraventricular nuclei (PVN) [85], both of which receive synaptic inputs from SCN AVP+ cells and play a causative role in the expression of light-mediated behaviour [84, 85]. The SCN is also known to project to the lateral septal region[86]―one of the gateways of the septohippocampal system―providing an additional pathway to regulate behavioural arousal to light [87]. (5) Finally, GluA1 subunits on astrocytes [88] may modulate neuronal activity directly via the release of glutamate onto neurons or indirectly by altering glial responses [47]. Given recent evidence for the importance of cellular clocks in astrocytes within the SCN [89], this provides an additional mechanism by which AMPA receptor subunits may influence SCN function. These various possibilities remain to be examined in future studies.

### GluA1 deficiency attenuates negative masking and SCN cFos induction without affecting circadian photoentrainment

Despite their attenuated SCN response to light, GluA1 knockouts’ phase advance under jet lag and phase delay of wheel-running activity by nocturnal light were indistinguishable from wild-types, suggesting that GluA1-mediated signalling is important for negative masking and SCN cFos induction but not circadian photoentrainment. This is at odds with the assumption that photic cFos induction in the SCN is closely related to phase shifting of the circadian clock [90]. It is also inconsistent with the findings from a previous study, which demonstrated the involvement of AMPA receptors in photoentrainment [43]. It reported that local infusion of AMPA into the mouse SCN in vivo during the subjective evening caused a phase delay in wheel-running activity, an effect that was eliminated by coadministration of AMPA and AMPA receptor antagonist; in addition, application of AMPA on cultured SCN at different circadian times induced phase delay and advance of *Per1*-*luc* bioluminescence signals in vitro, reproducing the typical phase response curve (PRC) of the mouse [43]. Apart from GluA1, GluA2–4 proteins and mRNA are expressed in the SCN [42, 43]. Although the subunit composition of synaptic AMPA receptors in the SCN remains to be determined, non-GluA1-containing AMPA receptors may support photoentrainment in GluA1-knockout animals; NMDA-type glutamate receptors could also play a complementary role in photoentrainment [91]. Furthermore, our data suggest that negative masking and phase-shifting responses―both are known to involve the retina→SCN pathway [92]―can be partially dissociated from each other in certain cases.

### Aberrant responses to environmental cues

In addition to circadian disturbances, GluA1 knockouts showed elevated sensitivity to environmental lighting at dusk and dawn, despite the fact that they were exposed to these visual changes repeatedly every 12 h. Heightened sensitivity to recently encountered familiar stimuli was also observed, leading to impaired odour recognition memory performance―similar to the deficit found in the object recognition task [22]. These data extend and complement our previous finding of stimulus-specific sensitisation to recent experiences in GluA1 knockouts [23]. The failure to dampen attention allocated to mundane events is also related to the deficit in pre-pulse inhibition of acoustic startle responses in *Bdr* mice [81] and sensorimotor gating deficits in schizophrenic patients [93–99]. Collectively, these different behavioural phenotypes may stem from a common deficit in filtering-out irrelevant stimuli [100–102]―more recently termed aberrant salience [103]―resulting in unregulated and heightened attention allocated to mundane events. The link between GluA1 and aberrant salience has been demonstrated in our previous study [33]. In the absence of GluA1, mice show unregulated attentional processing of the environment, indicated by persistently heightened levels of θ-band (6–12 Hz) power in the dorsal hippocampus and prefrontal cortex [33]―which may have contributed to GluA1-knockout animals’ exaggerated behavioural arousal to recently encountered familiar cues in our current study. Aberrant salience may facilitate formation of inaccurate associations between trivial sensory inputs, internal states, or both [104, 105], and has long been suggested as one of the major contributors to psychotic (positive) symptoms of schizophrenia [103]. In our current study, aberrant salience of environmental cues could be the common basis that gives rise to both circadian and attentional abnormalities in GluA1 knockout mice.

Apart from aberrant processing in higher-order brain areas, GluA1 deficiency in olfactory regions may also contribute to impaired odour recognition memory performance. In neonatal rats the pairing of a novel odour stimulus with systemic injection of isoproterenol, a β-adrenoceptor agonist, is known to produce appetitively-conditioned odour preference. This type of odour learning increases GluA1 subunits in synaptoneurosomes isolated from the olfactory bulb [106]. In addition, local infusion of an interference peptide derived from the carboxyl tail of GluA1 subunits (Tat–GluA1_CT_) into the olfactory bulb impairs this appetitively-conditioned odour preference [106]. Thus, impaired short-term odour memory performance could be the result of altered GluA1-mediated signalling in multiple brain regions.

### Fixing the compromised biological clock

Similar to the comorbidity of sleep disruption, circadian misalignment, and psychotic and cognitive symptoms in patients [49, 71, 107–110], we found that circadian disruption and recognition memory impairment co-occurred in GluA1-deficient mice. Recent patient studies suggest that the extent of sleep and circadian disruption may precede the onset, as well as predict the severity, of psychotic and cognitive symptoms in clinical and healthy populations [108–112]. This raises the possibility that ameliorating sleep and circadian disruption may improve various symptoms of schizophrenia [113]. In fact, strengthening circadian rhythms may improve physiology and behaviour in a range of different mouse models of neurodegenerative and neuropsychiatric diseases. This can be achieved via: (1) scheduled access to running wheels [114, 115], (2) restricted nocturnal feeding [116, 117], (3) exposure to certain lighting regimes in the light phase [118, 119], or (4) normalisation of GluA1 levels [120]. An exception is the early growth response 3 (EGR3)-deficient mouse model, which shows enhanced circadian rhythmicity but reduced sleep [121] and impaired object recognition memory performance [122]. Interestingly, in a mouse model of neurodevelopmental disorder lesioning the SCN can rescue object recognition memory impairment [123], suggesting that being without a central clock is better than having a compromised biological clock [124]. The effectiveness of these different methods in improving circadian and behavioural abnormalities in GluA1-deficient mice awaits to be examined in future studies.

## Conclusion

Deletion of the *Gria1* gene causes circadian disturbances similar to those reported in schizophrenia (Table 3). These circadian disturbances could be the result of altered AMPA-receptor-mediated signalling at multiple levels along the retina→SCN pathway. In addition, mice lacking GluA1 showed elevated attention to recently encountered familiar environmental stimuli, corresponding to the sensory gating deficit widely reported in patients. Impaired short-term odour memory process could be the result of aberrant processing in olfactory regions in addition to higher-order brain regions (e.g., the hippocampus). We hypothesise that aberrant salience could be the common basis by which AMPA receptor dysfunction gives rise to both circadian and attentional abnormalities in GluA1-deficient mice.

## Supplementary information

Supplementary Fig. S1
