# Supplementary material for: Deletion of AMPA receptor GluA1 subunit gene (Gria1) causes circadian rhythm disruption and aberrant responses to environmental cues
Source: Transl Psychiatry. 2021 Nov 15;11:588. doi: 10.1038/s41398-021-01690-3 (PMC8593011; doi:10.1038/s41398-021-01690-3)
Supplement: Supplementary file 1 — Supplementary Fig. S1 [file 41398_2021_1690_MOESM1_ESM.docx]

**
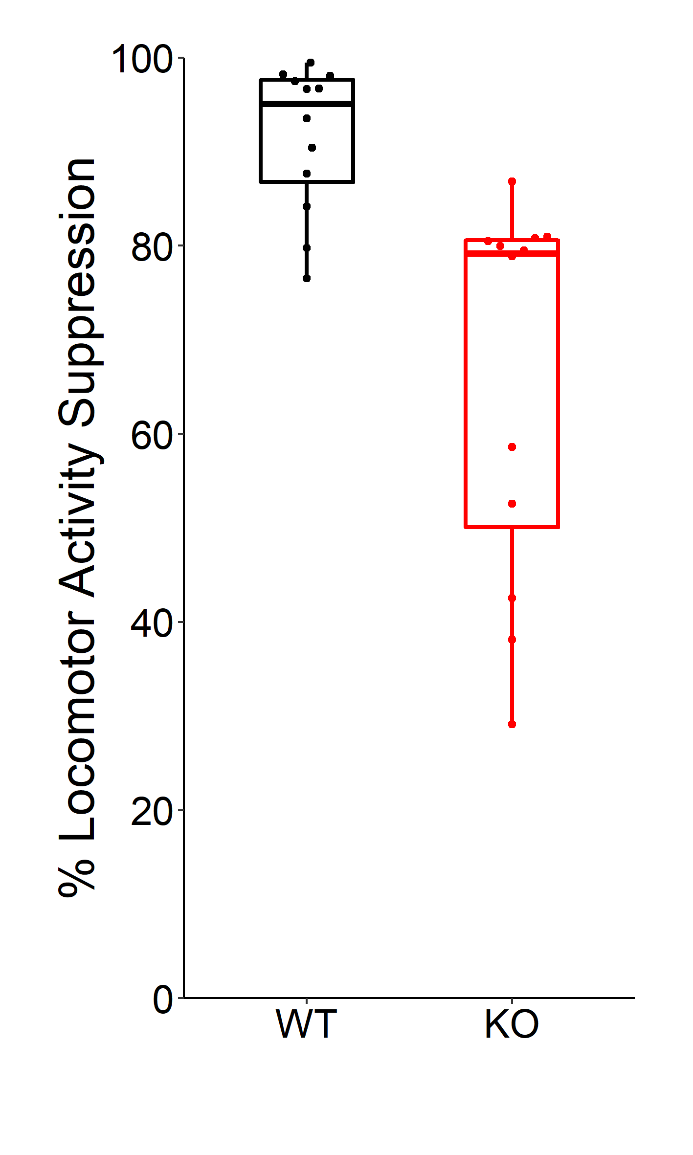
**

**Suppl. Fig. S1.** Nocturnal-light-induced suppression of locomotor activity (*negative masking*) as measured by video tracking was attenuated in GluA1-knockout animals (one-way ANOVA, *p* < 0.001). In addition, the variability in the GluA1-knockout group was higher than that in the wild-type group (Levene’s test for equality of variances, *p* < 0.001). n = 12 mice per genotype.
